# Enhancing Image Reconstruction Method in High-Frequency Electric Field Visualization Systems Using a Polarized Light Image Sensor

**DOI:** 10.3390/s25051596

**Published:** 2025-03-05

**Authors:** Kiyotaka Sasagawa, Ryoma Okada, Maya Mizuno, Hironari Takehara, Makito Haruta, Hiroyuki Tashiro, Jun Ohta

**Affiliations:** 1 Division of Materials Science, Graduate School of Science and Technology, Nara Institute of Science and Technology, 8916-5 Takayama, Ikoma 630-0192, Nara, Japan; okada.ryoma.on9@ms.naist.jp (R.O.); t-hironari@ms.naist.jp (H.T.); tashiro@ms.naist.jp (H.T.); ohta@ms.naist.jp (J.O.); 2 Medilux Research Center, Nara Institute of Science and Technology, 8916-5 Takayama, Ikoma 630-0192, Nara, Japan; 3 Radio Research Institute, National Institute of Information and Communications Technology, 4-2-1 Nukuikitamachi, Koganei 184-8795, Tokyo, Japan; mmizuno@nict.go.jp; 4 Department of Opto-Electronic System Engineering, Chitose Institute of Science and Technology, 758-65 Bibi, Chitose 066-8655, Hokkaido, Japan; m-haruta@photon.chitose.ac.jp; 5 Institute for Research Initiatives, Nara Institute of Science and Technology, 8916-5 Takayama, Ikoma 630-0192, Nara, Japan; 6 Department of Health Sciences, Faculty of Medical Saaaciences, Kyushu University, 3-1-1 Maidashi, Higashi-ku, Fukuoka 812-8582, Fukuoka, Japan

**Keywords:** electric-field imaging, electro-optic effect, image processing, image sensor, optical heterodyne

## Abstract

This paper introduces an image processing method, used to achieve uniform sensitivity across the imaging plane in a high-frequency electric field imaging system, that employs an electro-optical crystal and a polarization image sensor. The polarization pixels have two polarization directions, 0° and 90°, in pairs, and, conventionally, their difference is computed first. In contrast, this study proposes a method to separate each polarization image, perform pixel completion, and subsequently perform intensity correction. The proposed method was demonstrated to improve field distribution images acquired using 36 GHz and 30 GHz input signals for a microstrip line and patch antenna, respectively. From the measurement results of the microstrip line, the application of the proposed method reduced the electric field fluctuations on the line from 3.1 dB to 1.5 dB. This image-processing method can be applied sequentially during image acquisition, making it suitable for the real-time imaging of electric fields.

## 1. Introduction

Using electro-optic (EO) crystals for electric field measurement demonstrates a low level of invasiveness because it does not use any metallic materials for the probe [1,2,3,4]. Using light, high-frequency signals can be read out with low propagation loss, making it easy to handle high frequencies. Therefore, this method can be applied to high-frequency measurements in the millimeter-wave band and near THz [5], which have recently seen significant attention in research [6,7,8,9].

The low invasiveness of EO crystals enables an increase in the crystal size. A method was proposed to accelerate imaging by using this principle for optical scanning [10] or by integrating imaging optics with an image sensor to perform the two-dimensional batch measurement. This leads to a significant speed-up. Additionally, this method can obtain information on electric field intensity and phase. A two-dimensional EO sampling technique using an ultrashort pulsed laser and image sensor was reported by Wu and Zhang et al. and later developed by other groups [5,11,12,13,14,15,16]. While image sensors typically exhibit low resolution for detecting light intensity variations, this method could detect signals by generating relatively significant intensity changes. However, acquiring a specific frequency signal required adjusting the retroreflector, sampling the data several times, and obtaining the spectrum by performing a Fourier transformation. This poses challenges for achieving rapid measurements.

**Figure 1:**
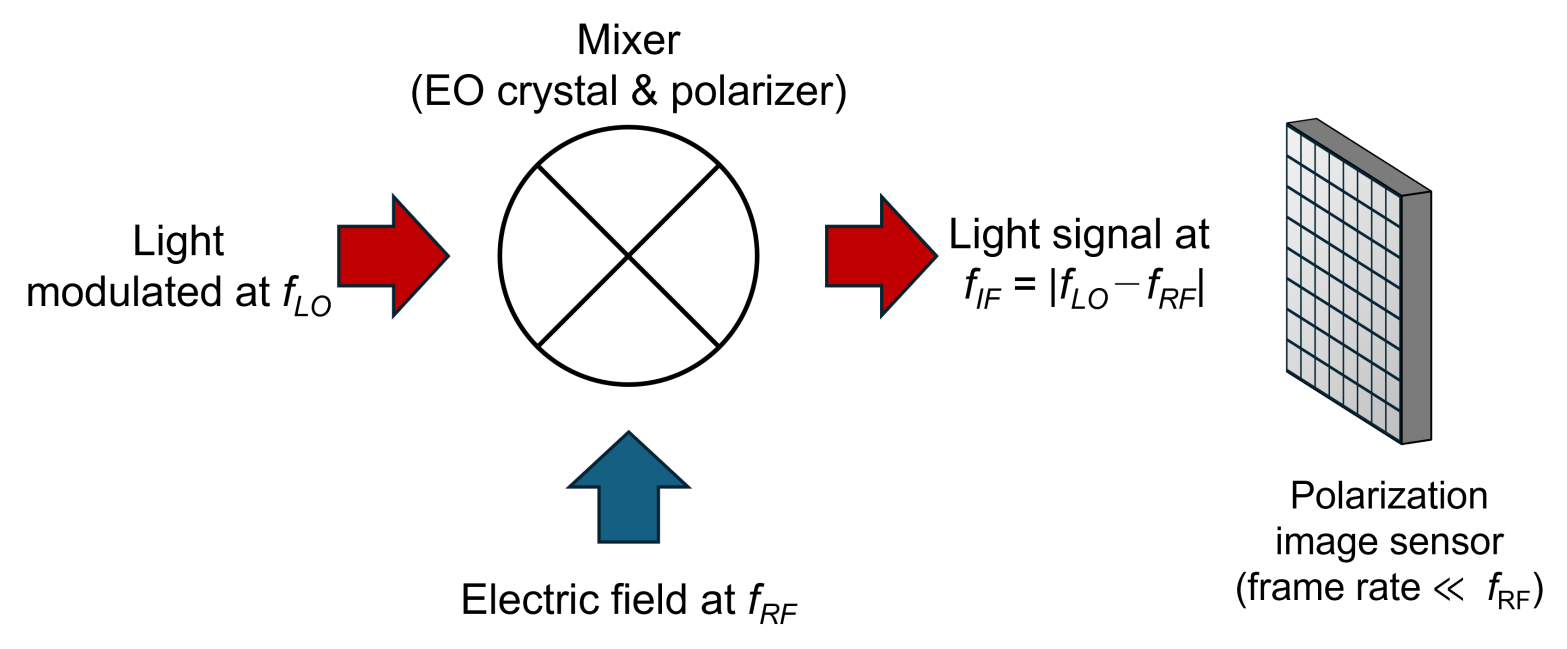
Schematic illustrating frequency conversion using optical heterodyne detection.

The optical heterodyne method is more suitable for acquiring electric field signals only at specific frequencies in a relatively short time [17]. This method has been typically reported in the microwave band; however, techniques for generating modulated light at high frequencies in a stable manner have been established recently, along with extensions to the THz band [18]. However, in such cases, the optical signal variation with field intensity becomes weak. One solution is improving the signal-to-noise ratio. Hence, this study proposes a configuration using a polarization image sensor, as shown in Figure 1 [19]. In this method, the irradiated light is modulated at a frequency slightly different from the electric field of the observed object and mixed with the electric field signal using an EO crystal and a polarizer. This is differentially detected using a polarization image sensor that can separate and detect mutually orthogonal polarization components to reduce phase noise. Here, an originally designed polarization image sensor with a large pixel capacity [20] is employed because general image sensors are not well-suited to read out such weakly varying signals [21]. Furthermore, a field imaging system with a double polarizer configuration is proposed as the optical system. These methods avoid challenges in image sensor utilization, such as pixel saturation, low-intensity resolution, and the inability to perform differential detection with a simple device system. These methods significantly improve the sensitivity of field imaging.

As mentioned earlier, parallelization using image sensors leads to significant improvements in speed. However, it is affected by in-plane non-uniformity. Regarding high-speed imaging, real-time imaging is expected to be applied to device evaluation on the manufacturing line. However, in-plane sensitivity non-uniformity has been one of the issues. For this, one goal is to achieve an accuracy of about ±1 dB, which is the accuracy of a general spectrum analyzer. For example, if there is a birefringence in the crystal, it can be effectively compensated using a waveplate in single-point measurements. Similarly, if the coupling efficiency to the photodetector deviates from the optimum point, it can be easily canceled if the deviation is identical throughout the measurement. In contrast, inhomogeneities in the in-plane characteristics appear in systems using image sensors. Specifically, in the double polarizer configuration, the transmitted light is detected in a cross-Nicol arrangement, resembling a dark-field optical system. Consequently, the detected light intensity is much lower than the incident light intensity, and the in-plane characteristic distribution of the optical system is significantly affected.

This study describes a method to reduce artifacts caused by such in-plane characteristic variations through image processing. Specifically, it proposes and demonstrates a technique to reduce the spatial and temporal deviations in the detection sensitivity by improving the process of generating electric field distribution images using simple signal processing, as used in previous reports. Additionally, a method is introduced to enhance spatial resolution, achieving half the pixel count of conventional electric field distribution generation. Section 2 describes the principles of a device that visualizes electric fields using a polarized light image sensor and electro-optic crystals. Section 3 explains the proposed method for extracting electric field distribution images. Section 4 demonstrates that the proposed method reduces noise and improves the spatial resolution of the electric field distribution image by comparing actual imaging examples with the conventional method. Section 5 explores the possibility of real-time implementation and pre-processing on the image sensor chip, and Section 6 presents the significant conclusions of the study.

## 2. Principle of Signal Acquisition for Electric Field Visualization System Using a Polarized Light Image Sensor

### 2.1. Electric Field Measurement Using Electro-Optic Effect

Refractive index modulation due to the Pockels effect is read using light. This is discussed in detail for zinc-blend structures, such as ZnTe, the EO crystal used in the proposed system. When an electric field is applied, the refractive index changes, as follows:

(1) $$\begin{array}{c} \Delta n=\pm \frac{1}{2}n^{3}r_{41}E, \end{array}$$

where *n* is the refractive index of the medium (EO crystal) and $r_{41}$ is the nonlinear optical coefficient. The phase of the transmitted light changes according to the refractive index. For example, ZnTe has no birefringence when no electric field is applied. Conversely, when an electric field is applied, the sign of the refractive index change differs depending on the direction of polarization, resulting in a phase change in polarization. When light is transmitted through ZnTe, the polarization state varies in accordance with the intensity of the electric field.

To be measured by a photodetector, the signal must be in the form of light intensity information. While photodiodes are widely used as high-sensitivity photodetectors, they are inherently not polarization-responsive. Therefore, the phase change information must be converted into intensity variations. This can be achieved by inducing light interference. There are two possible methods for this, as follows: one is to use a resonator such as a Fabry–Perot resonator [22,23,24,25,26], and the other is to use polarization interference. The advantage of using a resonator is high levels of sensitivity detection using the resonance effect. Application to the millimeter wave band used for 5G was also reported, although the frequency band is limited [26]. When the wavelength of the irradiated light is aligned with the resonance peak, and the output light intensity is measured, the refractive index change causes the resonance wavelength to shift and the output intensity to change. The higher the *Q*-value of the resonator, the greater the detection sensitivity. However, an increase in the *Q*-value narrows the line width of the resonance frequency and the bandwidth of the frequency that can respond.

Meanwhile, polarization interference uses a polarizer as an optical analyzer. For two polarization components that are orthogonal to each other, the polarizer is positioned so that the direction of transmitted polarization is ±45 degrees with respect to both. If there is a phase difference between the two polarization components, the result of the interference is obtained as the transmitted light component. This technique does not use the resonance effect, and therefore has a wide bandwidth but low sensitivity.

In electric field imaging systems using image sensors, the information on refractive index changes is converted into information on intensity variations and is detected using polarization interference. This is because there is little influence of the inhomogeneity of the in-plane characteristics on the EO probe, making it easy to create a system to measure in-plane distribution using an image sensor.

### 2.2. Optical System

**Figure 2:**
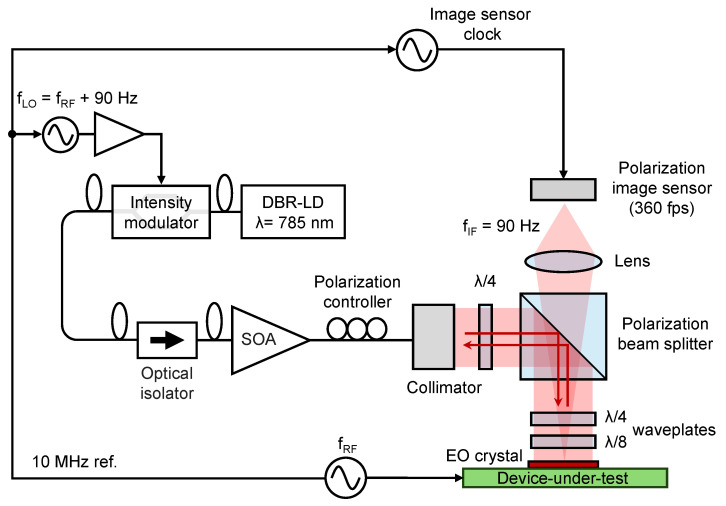
Electric field imaging system setup.

The optical system uses a dual polarizer structure that was recently developed, as shown in Figure 2 [19]. This technique utilizes a polarization image sensor that detects two mutually orthogonal polarization directions and a uniform polarizer. The uniform polarizer selectively transmits the polarization component produced because of the birefringence change in the field probe, and the incident linear polarization is significantly reduced. This results in a decrease in light intensity and an increase in modulation.

The EO crystal used in the experiments in this paper is (100)-ZnTe, with dimensions of 3 mm × 3 mm × 0.3 mm. For the light source wavelength of 780 nm, an antireflection coating is applied on the top surface and a high reflection coating on the bottom surface.

A polarizing beam splitter (PBS) is used as the uniform polarizer. The polarizers for the pixels are realized using the wiring layer of the CMOS process used for image sensor fabrication. The extinction ratio of the former is approximately 800, while that of the on-pixel polarizer is approximately 3.

The PBS is in a cross-Nicol arrangement. Most of the incident linearly polarized light is reflected by the PBS. However, the polarization component, which is generated by applying an electric field to the EO crystal, is almost completely unreflected and transmitted. Thus, the light intensity is reduced by the PBS while the modulation is increased.

The polarizers in the optical system are used to adjust the polarization state. A quarter-wave plate on the incident side is used to adjust the polarization to a highly accurate linear polarization, and a pair of quarter-wave and eighth-wave plates between the PBS and the EO crystal provide a retardation of half wavelength and quarter wavelength, respectively, as the light passes twice. These wave plates are adjusted to maximize the reflection by the PBS.

The focal length of the lens is 50 mm and the magnification of the image from the EO crystal to the lens is approximately one. The optical resolution of this system is determined using the pixel size of the image sensor (30 µm).

The signals transmitted through the polarizer on the pixel are complementary to each other. Due to the change in the refractive index of the electro-optic crystal, an increase in one causes a decrease in the other. This characteristic is utilized to compute the difference between the two sides and perform differential detection. In this process, the signal components are added, and the phase noise is reduced. This phase noise is external, from sources such as light source intensity fluctuations and vibrations, and, as a result, the signal-to-noise ratio is improved. This method increases the sensitivity but reduces the linearity, resulting in a narrower dynamic range. However, the signal degradation due to image sensor pixel limitation can be avoided. Conversely, while the dual polarizer configuration also reduces the dynamic range, this is not a major issue in electric field measurements because the change is minimal.

### 2.3. Image Sensor Used in the System

**Figure 3:**
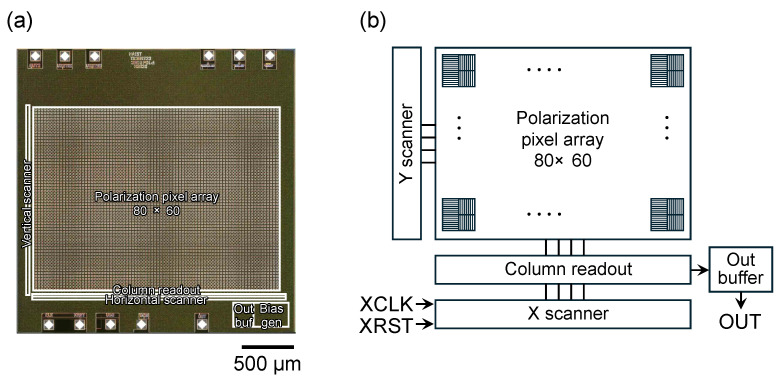
(**a**) Image and (**b**) block diagram of the polarization image sensor.

In this study, a polarization image sensor is used, which allows batch differential detection in the imaging plane and reduces noise in-phase. The image sensor was the same as those used in the past [27]. Each row contains pixels with polarizers in an orthogonal relationship to each other. An image of the actual chip is shown in Figure 3, and the specifications are given in Table 1. The number of pixels is 80 × 60, but there are 40 × 60 polarizer pairs in each pixel. Various methods have been created for the fabrication of on-pixel polarizers [28,29,30,31,32,33,34,35,36,37,38]. In this sensor, the polarizer is a grating structure composed of two metal interconnected layers, within the 0.35 µm CMOS process used for fabrication. The lines of and space between the gratings as polarizers are 0.7 µm and 0.7 µm, respectively, which is larger than the wavelength. This polarizer does not have a high extinction ratio; however, it does provide the characteristics of a polarizer. Polarization image sensors fabricated using dedicated CMOS processes demonstrate a higher extinction ratio [39,40]; moreover, some EO sampling systems have been reported to be using such polarization image sensors [41,42]. However, even if the extinction ratio is nearly infinity, the improvement ratio is approximately twice as high for our sensor [27]. In addition, because of the rolling shutter, the timing of signal acquisition varies across rows, resulting in artifacts when acquiring phase information. However, as discussed below, for the present application, phase correction can be applied to the obtained results to avoid this effect. The output from the image sensor is an analog voltage output, which is converted to a digital signal using a 14-bit analog-to-digital converter. The frame rate of the image sensor was set to 360 fps due to the limitations of the system used in this study. The intermediate frequency was set at 90 Hz, which is a quarter of that rate.

### 2.4. Effect of Noise

The modulation signal amplitude acquired in EO detection is proportional to the light intensity. Hence, if the light intensity fluctuates in time or space, it can become a noise component. The light intensity also varies with time, and, although it is stabilized, it still exhibits slight variations. Moreover, it depends on whether the driver maintains a constant current or a constant light intensity, such as when using a laser light source. In short-term observations, a constant current source is more stable, but over the long term, light intensity drift is more likely to occur. This study uses a laser diode set to constant current as the light source. In the case of pixel arrays, achieving perfectly uniform in-plane conditions is challenging. Light intensity and polarization vary slightly from pixel to pixel.

As mentioned earlier, differential acquisition between different polarization components helps reduce in-phase noise. However, the enhancement achieved by adding signal components is not very sensitive to the differences between them. Moreover, the signal levels must be equal for effective in-phase noise reduction.

Image sensors generally store photo carriers in the capacitance of a pixel; however, the pixels have saturation limits. As a result, pixel saturation easily occurs when exposed to strong light. Additionally, when light is sufficiently intense, the signal-to-noise ratio is primarily limited by photon shot noise, which dominates the noise component. In other words, a signal that is easy to measure with an image sensor is one that reduces light intensity and avoids pixel saturation while simultaneously increasing the signal-to-noise ratio. The dual-polarizer structure achieves this. The EO signal is proportional to the light intensity; hence, as the light intensity decreases, the signal decreases. However, under photon-shot noise-limited conditions, the noise decreases in proportion to the square root of the light intensity. The signal enhancement ratio due to the dual polarizer structure is the square root of the light intensity irradiating the EO crystal. Thus, the SNR is comparable to that of the detection system, assuming that pixel saturation does not occur. Although the dynamic range is reduced, this is not a significant challenge in electric field measurements because the change is minimal.

## 3. Proposed Image Processing Method

**Figure 4:**
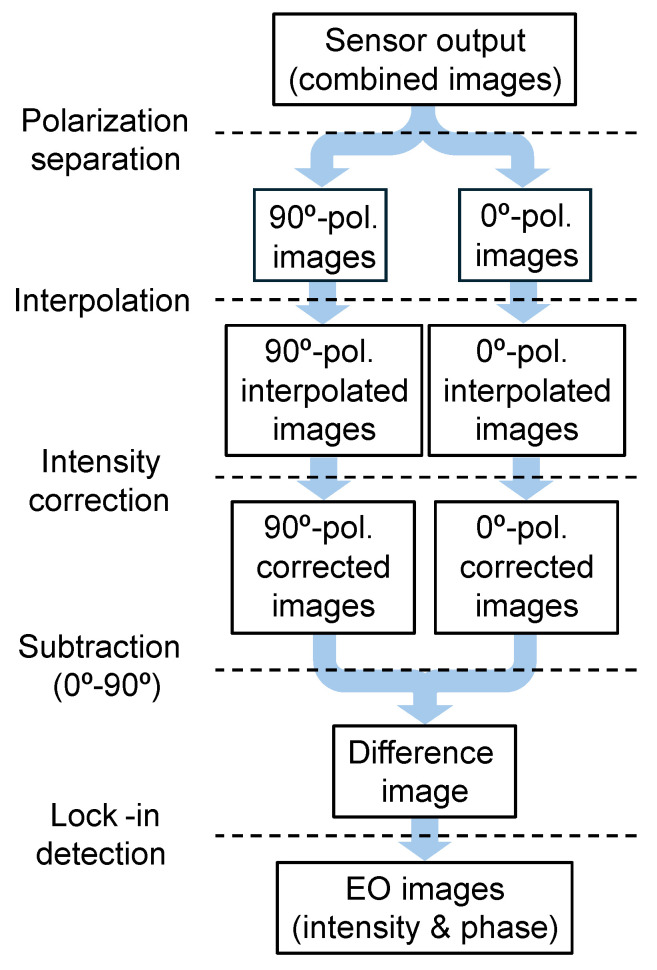
Flowchart of image processing using the proposed method.

The flowchart for the proposed image processing is shown in Figure 4. After separating the two polarization images, image completion is performed for each of them. Using this image, intensity correction is performed to obtain a difference image. Furthermore, the intermediate frequency components are extracted from them to obtain intensity and phase information. The details of each process are described below.

### 3.1. Generation of Each Polarization Image

Ideally, when differential detection is used, it is desirable to obtain two types of polarization information that are orthogonal to each other at the same point. However, in polarization image sensors, each polarization pixel performs signal detection at a different position. Conventionally, the spatial resolution has previously been found to be low because of the difference between a pair. In contrast, in this study, after being separated into their respective polarization images, the columns in between are complemented using an interpolation method with magnification factor of 2. This reduces the reduction in spatial resolution. One of the most common completion methods, the bicubic method, allows for higher precision than generating simple, intermediate values.

### 3.2. Intensity Correction

The electric field signal is read as the amplitude of the intermediate frequency component in the image sensor detection signal. Because this amplitude varies with light intensity, it is desirable to irradiate as uniform light to reduce errors among pixels. However, in reality, the light intensity distribution varies depending on various factors. Especially in the case of a dual polarizer configuration, slight differences in characteristics appear as large variations, although they are significantly reduced by the uniform polarizer. To minimize this effect, each pixel value is divided by the average value of the 100 frames immediately preceding the acquired image.

### 3.3. Difference Acquisition

Since an almost standardized image was generated in the previous process, the difference between the two polarization images is simply acquired here.

### 3.4. Intermediate Frequency Extraction

Software lock-in detection is used to extract the intermediate frequency [43]. In this study, as in our previous studies, the intermediate frequency is set to a quarter of the frame rate, and a set of four frames is used for calculation. The product of the acquired image data and the intermediate frequency, which is the reference waveform, is obtained and integrated. Here, the actual multiplication is not performed; it is realized using the operations of addition and subtraction, as follows:

(2) $$\begin{array}{cc} S_{inphase} & =\sum_{n=0}^{(N-1)/4}\left(I_{4n+1}+I_{4n+2}-I_{4n+3}-I_{4n+4}\right) \end{array}$$

(3) $$\begin{array}{cc} S_{quad} & =\sum_{n=0}^{(N-1)/4}\left(I_{4n+1}-I_{4n+2}-I_{4n+3}+I_{4n+4}\right) \end{array}$$

where $I_{m}$ is the image with a frame number *m*, *n* is the set number for a set of four frames, and *N* is the number of all frames. Since one frame is a quarter cycle of the intermediate frequency, $S_{inphase}$ and $S_{quad}$ are the in-phase and quadrature components of the phase detection, respectively. From these results, the field intensity and phase are given by the following equation:

(4) $$\begin{array}{cc} {\left|E\right|}^{2} & \propto S_{inphase}^{2}+S_{quadrature}^{2} \end{array}$$

(5) $$\begin{array}{cc} \phi & =\arctan\frac{S_{inphase}}{S_{quadrature}}. \end{array}$$

As mentioned earlier, the image sensor used in this study is a rolling shutter type, causing a phase shift by the same degree across each row. The phase shift is corrected accordingly from the exposure timing difference between the rows. The advantage of this method lies in its simplicity for digital processing using a field programmable gate array (FPGA), etc. Specifically, the process involves the following steps: (1) multiplying with the reference frequency component and (2) performing low-pass filtering through addition and subtraction.

## 4. Imaging Demonstration

### 4.1. Images of Each Processing Procedure

**Figure 5:**
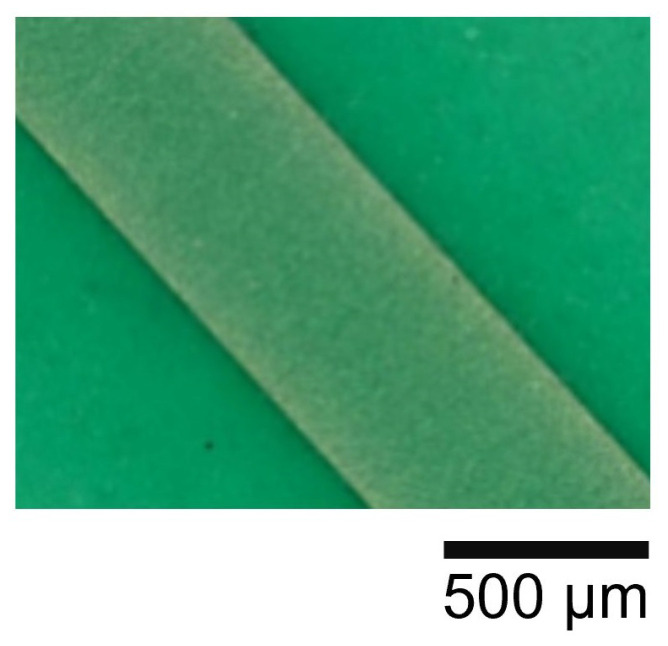
Image of the microstrip line.

A signal with a frequency of 36 GHz was inputted to the microstrip line, which was used as a device-under-test, and an EO crystal plate was placed on top of it to image the electric field distribution in the vicinity. Figure 5 shows a photograph of the microstrip line used in the experiment. A (100)-ZnTe crystal was used as the EO crystal. The bottom surface was HR-coated, while the top surface was AR-coated, creating reflective optics. The crystal was sensitive in the direction perpendicular to the crystal plane.

**Figure 6:**
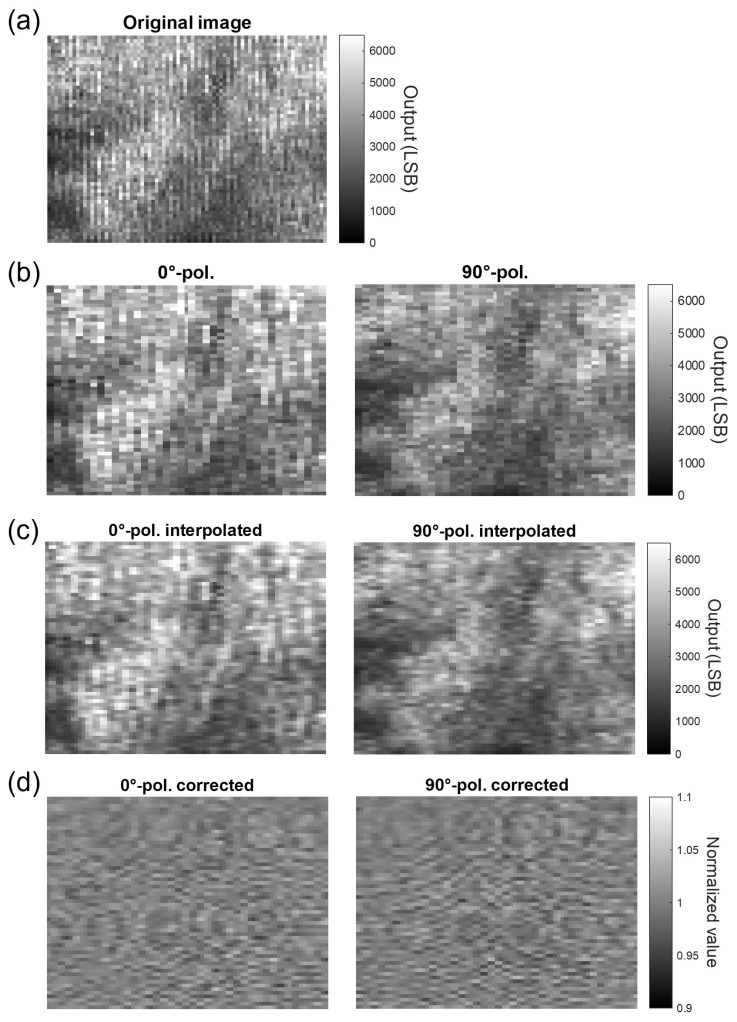
Examples of processing results for each of the proposed image processing steps.

Figure 6 shows acquired optical image examples from each step. The output light from a single optical fiber was irradiated by a beam expander. Ideally, the image should be smooth. However, as shown in Figure 6a, the brightness values varied slightly depending on the direction of polarization, resulting in a vertical stripe pattern. The refractive index change of the electro-optic crystal is slight, and the electric field distribution cannot be determined using this image alone.

Figure 6b shows the images separated by polarization direction. It can be seen that there is a difference between the two, although a correlation exists depending on the polarization direction. Figure 6c results from pixel completion using the bicubic method. This was used for subsequent processing. Figure 6d shows the results when the average of the previous 100 frames was used. It can be seen that, without intensity correction, there was a significant difference in the optical intensity between the bright and the dark areas, but this difference was significantly reduced. Consequently, an interference pattern appears in the image, attributed to the high coherence of the light source, which causes interference fringes even with slight contamination on the optical elements.

**Figure 7:**
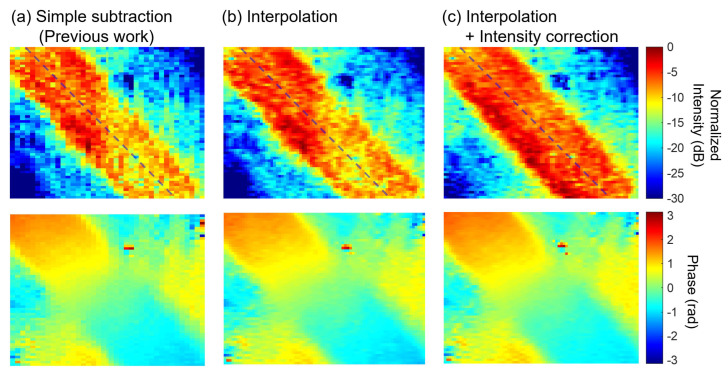
Examples of field intensity and phase images on a microstrip line. (**a**) Result of simple processing used in the previous work. (**b**) Result of applying column interpolation processing. (**c**) Result of applying column interpolation and intensity correction processing. The number of images was 65,536.

The results obtained by extracting the 90 Hz component are shown in Figure 7. The number of integrated images was 65,536. For comparison, the results from the conventional procedure, the results without intensity correction, and the results from the proposed method are shown side by side. Both results show that the electric field is concentrated just above the microstrip line. In addition, the phase gradually rotates as one moves forward in the line. A simple difference between conventional polarization pixel pairs shows that there are regions with significant differences across columns. This is because the optical intensity distribution is not exactly the same for different polarization directions, as shown in Figure 6b, and the component of the difference affects the intensity distribution. Conversely, when the images are complemented between columns, as in the proposed method, such differences in sensitivity between columns are reduced. Furthermore, the uniformity of the electric field intensity distribution on the microstrip line is enhanced by correcting the light intensity. In particular, the lower right portion of the acquired images shows that the light intensity is low even in Figure 6c, which is improved by the brightness correction.

**Figure 8:**
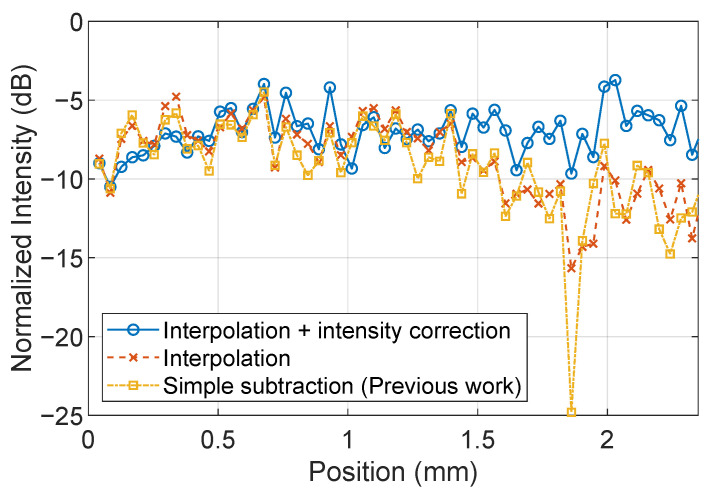
Intensity profile comparison on the solid blue lines shown in Figure 7.

Figure 8 shows the electric field intensity profile on the microstrip line shown in Figure 7. The electric field intensity on the microstrip line should ideally be constant, and it can be seen that the proposed method significantly improves it. Taking the standard deviation of the result, the electric field constant is 3.1 dB before correction, whereas it is 2.5 dB with image completion and further improved to 1.5 dB with luminance correction.

**Figure 9:**
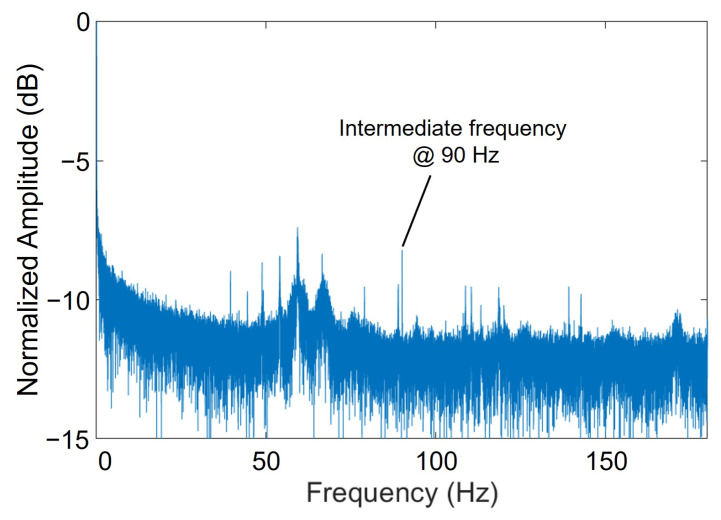
Spectrum at a point on the microstrip line track in the center of the image, calculated using FFT.

The time variation of the signal at the point on the microstrip line in the center of the image in Figure 9 was converted to a frequency spectrum by fast Fourier transform (FFT). A sharp peak is observed at 90 Hz, which is the intermediate frequency. Additionally, several other peaks are visible, some of which correspond to interference fringes. Of these noises, those near 60 Hz and its multiples originate in the power supply lines. At these low frequencies, they are also susceptible to vibration.

**Figure 10:**
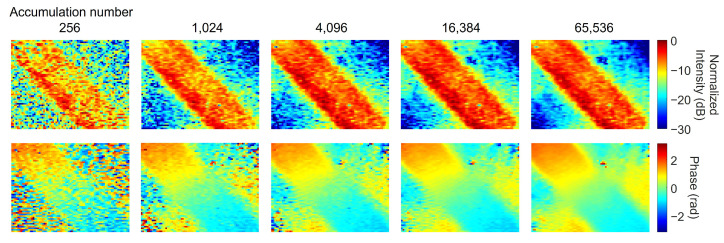
Comparison of images with different numbers of integrated images using signal extraction.

**Figure 11:**
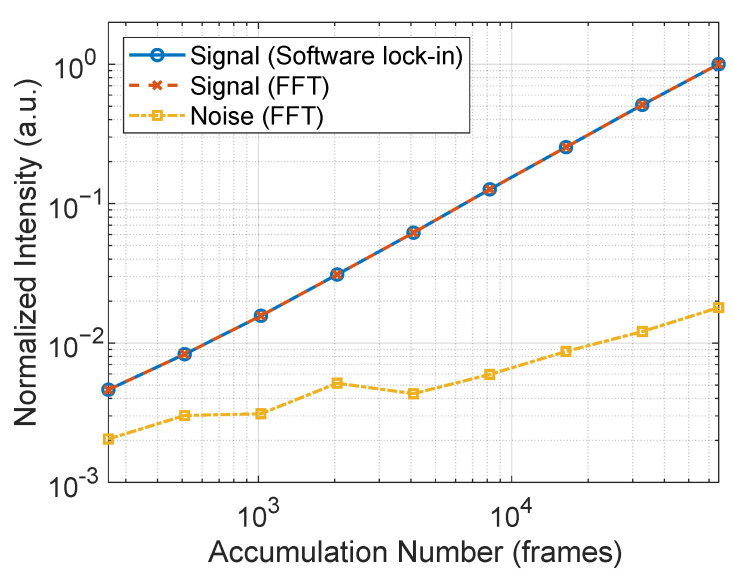
Dependence of signal intensity and noise components calculated from 5 × 5 pixels on a microstrip line on the number of integrated copies. Signal intensity is plotted as extracted by software lock-in detection and FFT. The noise is calculated as the average of 20 points in the region slightly removed from the intermediate frequency. All values are normalized with respect to the value at 65,536 frames.

Figure 10 shows the improvement in the field distribution image as the number of integrated frames increases, specifically showing a significant reduction in noise as the number of frames increases from 256. The signal on the microstrip line is large enough that further increases in frames no longer result in significant changes. Figure 11 plots the signal intensity, which is the average of 5 × 5 pixels at the center of the microstrip line normalized with respect to 65,536 frames. The signal intensity is proportional to the number of integrated frames, which is almost consistent with the theoretical improvement rate. Additionally, it is almost identical to the case using FFT, indicating that the software lock-in method shown in Section 3 provides a sufficient system. The FFT also shows the values of frequencies slightly removed from 90 Hz as noise components. From this figure, it can be inferred that, for 4096 or more images, the noise intensity is proportional to the square root of the number of images and follows a Poisson distribution.

**Figure 12:**
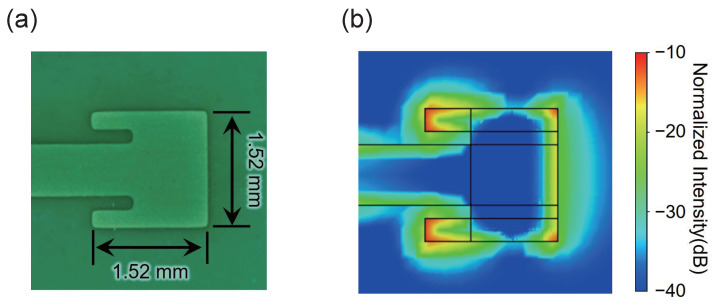
(**a**) Image of the 30 GHz patch antenna. (**b**) Simulation result of the electric field distribution perpendicular to the patch anntena.

**Figure 13:**
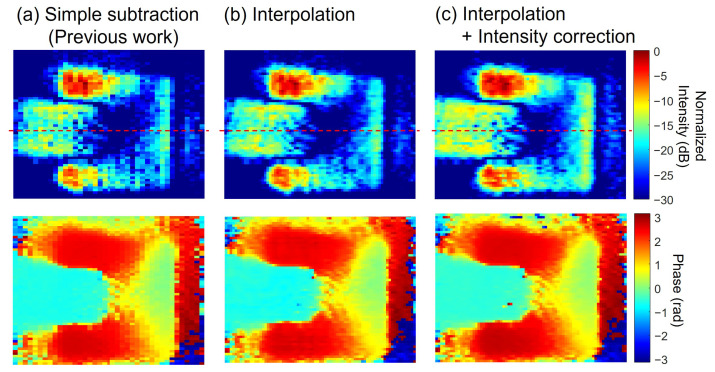
Examples of field intensity and phase images on a 30 GHz patch antenna. (**a**) Result of simple processing method used in the previous work. (**b**) Result after applying column interpolation processing method. (**c**) Result after applying column interpolation and intensity correction processing method. The number of images is 10,000 frames.

As an example of a different observation target, a patch antenna with a resonant frequency of 30 GHz was observed. The photograph of the antenna is shown in Figure 12a. The result of the simulation is shown in Figure 12b. The simulation shows the intensity of the component perpendicular to the plane at the surface portion of the patch antenna. It can be seen that the electric field is most strongly concentrated in the corner on the input side. As with the microstrip line, Figure 13 compares the results obtained using the previous method with the present results. These results also show improvement—reduced stripes in the row direction and enhanced flatness of the electric field distribution. From this comparison, it can also be seen that the spatial resolution in the horizontal direction has improved. Meanwhile, concentric interference fringes were observed to remain near the center of the image. Comparing these results with the simulation, there is agreement that the electric field is concentrated at the corners of the input side.

**Figure 14:**
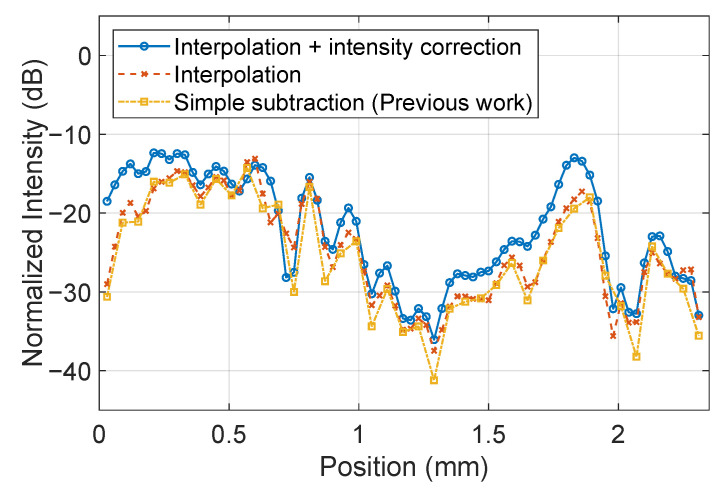
Electric field intensity profile on the line shown in Figure 13.

The intensity profile on the red dashed line in the center of Figure 13 is shown in Figure 14. This result also indicates that the difference in intensity can be corrected using the proposed method. The 3 dB width of the field concentration region at the right edge of the patch antenna is about 4 pixels, which roughly corresponds to 0.12 mm. In the simulation shown in Figure 12b, the spatial resolution is estimated to be about 0.06 mm. In this study, since 0.3 mm-thick ZnTe crystals were used, it is not possible to observe the distribution of electric fields at the edges. However, there are reports that finer structures can be observed by using thinner crystals, and this system is expected to provide similar improvements when high spatial resolution is required [44].

### 4.2. Movie Generation

**Figure 15:**
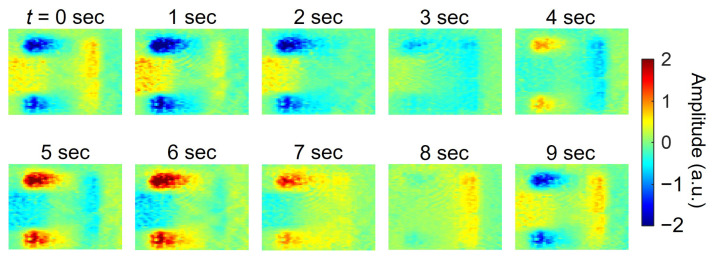
Results of continuous imaging and phase display of the electric field on a patch antenna rotating in phase with a 10 s period. The number of images is 360 frames.

The oscillation of the electric field can be visualized by displaying a phasor from the intensity and phase information. For the patch antenna in Figure 12, the frequency of the high-frequency electric field was shifted by 0.1 Hz from 30 GHz, the frequency of observation. This caused the phase to rotate within a 10-second period. Here, the phasor image was generated continuously from 84,960 frames of data, with the number of images integrated in each frame set to 360. Part of the result is shown in Figure 15. The results, shown as moving images together with intensity and phase images, are also shown in Supplementary Video S1.

These results clearly demonstrate that the electric fields on the right and left sides of the figure oscillate in opposite phases with each other.

## 5. Discussion

The proposed method improves the large signal variations that appear in conventional simple difference acquisition and improves the uniformity of sensitivity. The proposed method is more complex than the simple difference processing method between neighboring pixels used in our previous studies. Therefore, it is difficult to process in hardware. On the other hand, significant in-plane sensitivity variations are significantly reduced. However, there are still some residual noise components, including the noise component that appears as interference fringes. This component has been significantly reduced compared to the optical image shown in Figure 6. However, the standard deviation of 1.5 dB obtained from the profile on the microstrip line is still insufficient for the target and requires further improvement. One of the possible causes is because the noise component does not coincide with the intermediate frequency component of the observed object. Meanwhile, as shown in Figure 13, there are cases where the noise component remains after image processing. This is probably because some frequencies of the noise components may be superimposed on the detection target. To prevent this, the first step is to avoid the contamination that causes it, but this is not always possible. Another approach would be to move the intermediate frequency into a higher frequency band that is considered less noisy.

In addition, there are residual noise components that appear to be fixed patterns in the intensity image of the video and other data; while this could be due to contamination on the image sensor, it is also attributed to the non-uniformity of the characteristics of the electro-optic crystal itself, even after the contamination is removed. In such a case, it is necessary to base the correction on the characteristic distribution of the crystal. This can be achieved by obtaining correction information through observing a uniform electric field, for example.

While FFT requires the acquisition of all data before calculation, the software lock-in method used in this study can calculate up to the acquired frames and then add additional frames to obtain even more accurate results. It is thus suitable for real-time acquisition.

In the software lock-in method, it is necessary to obtain two types of results: in-phase and quadrature. Four frames are required to perform a set of calculations, but the sign combinations used to add and subtract each subframe are different for in-phase and quadrature. Performing these actions on the image sensor chip is expected to reduce the amount of data transmission and facilitate speed-up.

In the previous report, the signal-to-noise ratio was improved by differential amplification in the chip [45]. However, the image processing in this study is not applicable to such a chip because a completion process must be performed before taking the difference. When applying the proposed method in this study, the integration of each subframe is required on the hardware. If an appropriate implementation method can be determined, it is expected that real-time and high-sensitivity detection can be achieved by combining this method with dual polarizer structures and optical amplifiers.

## 6. Conclusions

A method was proposed to improve the electric field distribution image in a high-frequency electric field imaging system using a polarization image sensor. The method involves separating the image by polarization direction and performing pixel completion and brightness correction for each image. This method effectively reduces the noise component caused by columns and improves the non-uniformity of sensitivity distribution. In the verification using microstrip lines, in the area where the conventional method produced 3.1 dB of variation, the variation was reduced to 1.5 dB by using the proposed processing method. This method does not use FFT or other processing, relying only on sequential processing along with image acquisition, enabling real-time processing using hardware such as FPGA.

However, even with this method, some residual noise components remain. The measurement results in this study suggest several causes for this. Further improvements to the optics, image sensor, and other related components are expected to address these issues, resulting in higher-quality, low-noise field image imaging.

In this study, the observation of the near-field distribution on a high-frequency device was demonstrated using the proposed system, which can be used to evaluate the characteristics of such devices. Furthermore, it is expected to be applied to the evaluation of properties by irradiating high-frequency electromagnetic waves to an object and measuring its transmission and reflection characteristics.

## Figures and Tables

**Table 1:** Specifications of the polarization image sensor.

Technology	0.35 µm 2-poly 4-metal standard CMOS
Operating voltage (V)	3.3
Pixel number	80 × 60
Pixel type	3-transistor active pixel sensor
Pixel size (µm^2^)	30 × 30
Photodiode size (µm^2^)	15 × 15
Photodiode type	Nwell-Psub
Polarizer	Line/Space = 0.7 µm/0.7 µm (2 layers)
Extinction ratio	3.1 @ 780 nm
Maximum signal-to-noise ratio (dB)	55
Chip area (µm^2^)	2700 (W) × 2645 (L)

## Data Availability

The original contributions presented in this study are included in the article. Further inquiries can be directed to the corresponding author.

## Supplementary Materials

The following supporting information can be downloaded at: https://www.mdpi.com/article/10.3390/s25051596/s1, Video S1: Examples of field intensity and phase images.
